# Local Weather Station Design and Development for Cost-Effective Environmental Monitoring and Real-Time Data Sharing

**DOI:** 10.3390/s23229060

**Published:** 2023-11-09

**Authors:** Antonio Rivera, Pedro Ponce, Omar Mata, Arturo Molina, Alan Meier

**Affiliations:** 1 Institute of Advanced Materials for Sustainable Manufacturing, Tecnologico de Monterrey, Monterrey 14380, Mexico; a01337294@tec.mx (A.R.); omar.mata@tec.mx (O.M.); armolina@tec.mx (A.M.); 2 Lawrence Berkeley National Laboratory, 1 Cyclotron Road, Berkeley, CA 94720, USA; akmeier@lbl.gov

**Keywords:** weather analytics, sustainable monitoring, atmospheric data, predictive modeling, data collaboration, sensor networks

## Abstract

Current weather monitoring systems often remain out of reach for small-scale users and local communities due to their high costs and complexity. This paper addresses this significant issue by introducing a cost-effective, easy-to-use local weather station. Utilizing low-cost sensors, this weather station is a pivotal tool in making environmental monitoring more accessible and user-friendly, particularly for those with limited resources. It offers efficient in-site measurements of various environmental parameters, such as temperature, relative humidity, atmospheric pressure, carbon dioxide concentration, and particulate matter, including PM 1, PM 2.5, and PM 10. The findings demonstrate the station’s capability to monitor these variables remotely and provide forecasts with a high degree of accuracy, displaying an error margin of just 0.67%. Furthermore, the station’s use of the Autoregressive Integrated Moving Average (ARIMA) model enables short-term, reliable forecasts crucial for applications in agriculture, transportation, and air quality monitoring. Furthermore, the weather station’s open-source nature significantly enhances environmental monitoring accessibility for smaller users and encourages broader public data sharing. With this approach, crucial in addressing climate change challenges, the station empowers communities to make informed decisions based on real-time data. In designing and developing this low-cost, efficient monitoring system, this work provides a valuable blueprint for future advancements in environmental technologies, emphasizing sustainability. The proposed automatic weather station not only offers an economical solution for environmental monitoring but also features a user-friendly interface for seamless data communication between the sensor platform and end users. This system ensures the transmission of data through various web-based platforms, catering to users with diverse technical backgrounds. Furthermore, by leveraging historical data through the ARIMA model, the station enhances its utility in providing short-term forecasts and supporting critical decision-making processes across different sectors.

## 1. Introduction

Monitoring local environments is a crucial undertaking that enables a better understanding of climate change’s challenges and how to respond to them. Environmental changes usually refer to alterations in the natural surroundings of the Earth that can occur due to various factors such as human activities, climate change, natural disasters, and other natural processes. These changes can significantly impact the environment and affect many areas, including ecosystems, biodiversity, water resources, and human health [1]. One of the main areas affected by environmental changes is ecosystems. Ecosystems are complex systems of living organisms, their physical environment, and their interactions [2].

Environmental changes can disrupt the delicate balance of these systems and cause significant harm to plant and animal species and entire ecosystems. For example, deforestation can lead to habitat loss and threaten the survival of many species of plants and animals. At the same time, ocean acidification can harm marine ecosystems and disrupt the food chain [3]. Environmental changes also affect biodiversity; biodiversity refers to the variety of living organisms on Earth and the ecosystems they inhabit. Environmental changes such as habitat destruction, climate change, and pollution can lead to biodiversity loss, as species cannot adapt to the changing conditions [4]. This biodiversity loss can have far-reaching consequences, including the loss of ecosystem services such as pollination and water purification.

Water resources are also affected by environmental changes. Changes in temperature, rainfall patterns, and land use can affect the availability and quality of water resources. For example, climate change can lead to changes in rainfall patterns, which can affect water availability and lead to droughts and floods. Pollution from human activities can also contaminate water sources and affect the health of aquatic ecosystems. In addition, environmental changes can also have significant impacts on human health. Air pollution, for example, can cause respiratory problems, while exposure to chemicals and other pollutants can lead to long-term health problems such as cancer and reproductive issues [5]. Temperature and precipitation patterns can also affect the spread of diseases such as malaria and dengue fever [6,7]. As a result, environmental changes can significantly impact many areas, including ecosystems, biodiversity, water resources, and human health.

Addressing these changes and working towards sustainable solutions is essential to ensure a healthy and livable planet. Unfortunately, existing weather monitoring solutions are constrained by the high costs associated with sensors, digital systems, communication devices, power sources, trained personnel for maintenance and operation, limited coverage, and insufficient data sharing with the public [8]. These limitations hinder small users such as farmers, transportation companies, and local government agencies from accessing reliable and affordable weather data. Weather monitoring technology continually evolves to overcome these limitations and provide better user solutions.

The urgency for cost-effective weather monitoring is evident in today’s climate landscape. Such monitoring is pivotal, especially when considering the challenges of rapid environmental changes and the need for timely responses. The National Oceanic and Atmospheric Administration (NOAA) has been at the forefront of this, developing surface moorings equipped with cost-effective sensors to monitor air-sea interactions, carbon dioxide uptake, and upper ocean parameters, emphasizing the significance of real-time data sharing [9]. One significant trend in weather monitoring technology is wireless sensor networks (WSNs), a solution that is both affordable and efficient [10]. WSNs consist of small, low-power sensors distributed over a large area and communicate with a central base station to transmit data [11]. This technology collects high-resolution data in real time and reduces traditional weather monitoring solutions’ cost and maintenance requirements [12].

The evolution of data sharing, facilitated by advancements in both public and private online platforms, ensures that users are equipped with real-time insights, fostering informed decision-making across various sectors [13]. As a result, weather data can be used to develop applications that provide valuable insights and decision-making support for various industries. State-of-the-art weather monitoring technology continually improves with WSNs, IoT, AI, and data-sharing advancements [14]. These technologies are making it more accessible and affordable for small users to access reliable and accurate weather data, which is crucial for responding to the challenges of climate change.

Fixed stations are ground-based stations designed to receive and transmit signals to and from satellites. These stations typically have satellite communication systems, such as satellite television and internet services. Fixed stations are stationary and do not move, making them ideal for establishing a stable connection with satellites and long-term studies [15].

On the other hand, satellite images are pictures of the Earth taken from space using satellites. These images can be used for various purposes, such as weather forecasting, urban planning, and natural resource management. Satellite images are captured using satellite cameras orbiting the Earth [8].

To comprehensively address the complexities of environmental monitoring, it is essential to understand the diverse range of technological systems and methodologies employed in this field:

- **Fixed Stations:** Ground-based stations that receive and transmit signals to and from satellites.
- **Satellite Images:** Pictures of the Earth taken from space using satellite cameras.
- **Mobile Stations:** Stations that can move and are used in mobile satellite communication systems, such as satellite phones and GPS devices.
- **Remote Sensing:** The process of collecting data about the Earth from a distance, typically using satellites or aircraft. Remote sensing can be used for various applications, such as environmental monitoring and land use mapping.
- **Earth Observation:** Using satellites and other platforms to monitor and study the Earth’s surface, atmosphere, and oceans. Earth observation can be used for various purposes, such as weather forecasting, disaster response, and agriculture management.

Local weather stations provide accurate, real-time data for various applications, from agriculture to urban planning. However, with the rapid changes in environmental conditions and the increasing complexity of weather patterns, there’s a growing need for enhanced measurements and innovative monitoring techniques. Recent advancements, such as integrating machine learning with city buses, have shown promising results in real-time weather monitoring and prediction, emphasizing the importance of localized data collection [16]. Moreover, incorporating wireless sensor networks in agricultural management processes highlights the significance of tailored, on-field measurements for optimizing production and ensuring eco-sustainability [17].

This paper presents an innovative solution to address the abovementioned limitations by introducing a local open-source weather station (OSWS), a localized and affordable sensing platform. The OSWS is designed to monitor a range of environmental variables, including temperature, relative humidity, atmospheric pressure, CO_2_ concentration, and particulate matter (PM 1, PM 2.5, and PM 10), offering rapid and cost-effective on-site measurements. Moreover, the station possesses these variables and can forecast their values by leveraging historical data obtained through the employment of the Autoregressive Integrated Moving Average (ARIMA) model. The real-time monitoring capabilities of the OSWS play a crucial role in providing accurate data that aids decision-making processes in diverse domains such as agriculture, transportation, and air quality monitoring applications [18].

The primary objectives of this research revolve around developing and implementing an OSWS that offers a cost-effective solution for environmental monitoring, especially catering to small users. This paper aims to harness the open-source nature of the OSWS to enhance public data sharing and ensure seamless communication between the sensing platform and end users, ensuring that valuable data is readily available for making informed decisions and addressing environmental challenges stemming from climate change.

Furthermore, the design, development, and evaluation of this weather station serve as a model for future low-cost and efficient monitoring systems. Its affordability, accessibility, and real-time monitoring capabilities can significantly contribute to advancing sustainability efforts and mitigating the adverse effects of climate change. The WS’s interface with end users is crucial in facilitating effective data communication and enabling informed decision-making.

Overall, the affordability, accessibility, and real-time monitoring capabilities of the OSWS present a cost-effective solution for environmental monitoring and data sharing. The comprehensive approach in designing, developing, and evaluating this device establishes valuable information for developing similar low-cost and efficient monitoring systems, ultimately promoting sustainability and mitigating climate change impacts.

To offer a clear outline of the paper, the structure is as follows: Beginning with the Introduction in Section 1, the background and significance of the study are explored. Section 2 presents a thorough literature review, highlighting essential research and relevant developments. In Section 3, materials and methods are detailed and subdivided into Section 3.1, focusing on the design and development of the weather station, and Section 3.2, discussing data validation methods. Section 4 presents the results, further segmented into Section 4.1, showcasing the design results; Section 4.2, assessing the ergonomics; and Section 4.3, comparing the data. Section 5 delves into the discussion, analyzing and interpreting our findings. The paper culminates with the conclusions in Section 6, summarizing our research outcomes and their implications.

## 2. Literature Review

**Figure 1:**
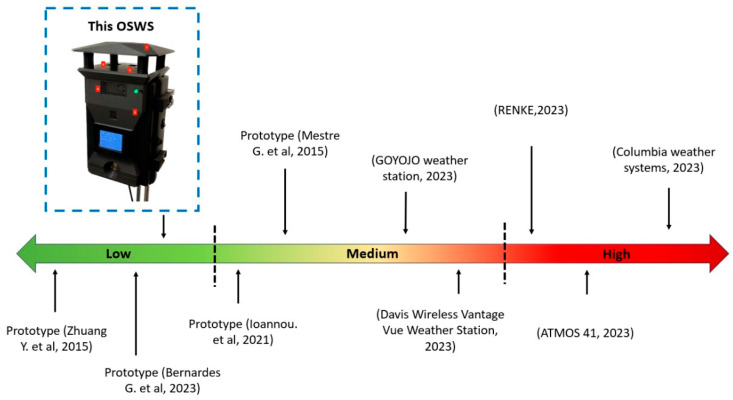
Cost comparison between various weather stations [19,20,21,22,23,24,25,26,27].

A systematic review was conducted to ensure a comprehensive understanding of low-cost local weather prediction. The search strategy targeted reputable scientific journals, querying various databases focusing on articles emphasizing innovative weather monitoring techniques or devices. The selection criteria were refined to prioritize peer-reviewed journal articles that discussed the challenges and potential of cost-effective and localized weather prediction methods. After identifying the relevant journal papers, a meticulous data analysis method was employed. This involved extracting and synthesizing data related to the efficacy, cost implications, and materials used (Table 1). The synthesized data provided insights into the advancements and challenges in low-cost local weather prediction. Figure 1 depicts a cost comparison between commercial weather stations, prototypes, and the OSWS presented in this paper.

## 3. Materials and Methods

**Figure 2:**
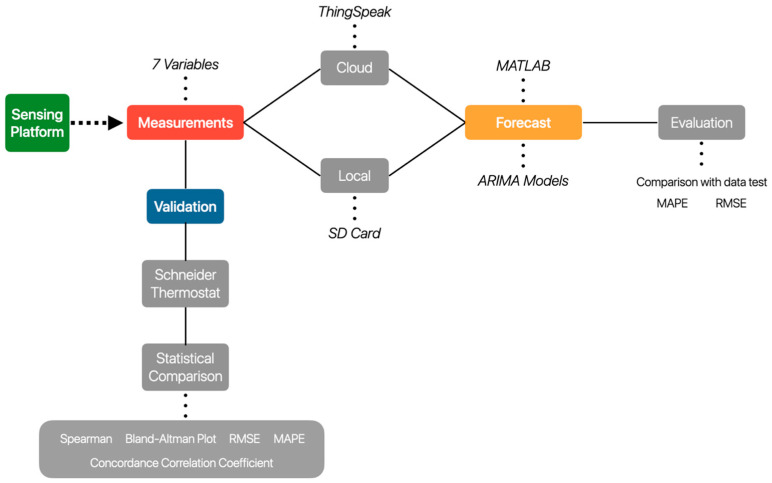
The general methodology.

The design steps deployed in this study are depicted in Figure 2, providing an overview of the research process. The initial stage, denoted by the green block, encompasses developing and fabricating the sensing platform for measuring environmental variables such as air temperature, relative humidity, atmospheric pressure, CO_2_ concentration, and particulate matter concentration (PM 1, PM 2.5, PM 10). During this phase, the information the WMO provided was used. This international agency ensures high-quality data delivery to end users.

Subsequently, once data for all variables were acquired, a comparison was conducted against commercial products to assess the proximity of the proposed station’s measurements to those of specialized devices. For indoor comparisons, a Schneider thermostat was chosen as the reference tool, while for outdoor comparisons, a Purple Air sensor was employed. It is crucial to emphasize that the reference sensors, although not entirely immune to measurement errors, were selected based on their use in industry and market availability and their certifications guaranteeing minimal errors. Consequently, the comparison focused on evaluating the monotonic relationship between the OSWS and these devices and determining errors (RMSE and MAPE) between them.

Moreover, to facilitate data storage, local storage (SD Card) and remote storage (ThingSpeak server (https://thingspeak.com/, accessed on 3 October 2023)) were implemented to capture and retain all collected sensor data. An ARIMA forecast model was also applied to predict values within a short-term timeframe using historical data. Notably, the model employed was univariate, considering only one variable per model. Thus, the time series containing values of a single variable (e.g., temperature) exclusively predicted values of that same variable. Finally, the forecast model underwent evaluation and comparison against known data, resulting in calculating parameters to assess its performance.

### 3.1. Weather Station Design and Development

The documents from the World Meteorological Organization have been followed to design a robust and accurate weather station as much as possible. There is a category called Automated Weather Stations (AWSs), and the current proposal fits into the multi-purpose AWS category. The main feature of these devices is that they measure, store, and transmit the data collected automatically [14]. Additionally, they must have at least three parts:

1. Sensing instruments.
2. Local modem or interface for a network connection.
3. Central processing unit.

Nevertheless, some other parts were added to the project to enhance their usability and performance. The features included were:

1. Power Sources.
2. Human-Machine Interface.

**Figure 3:**
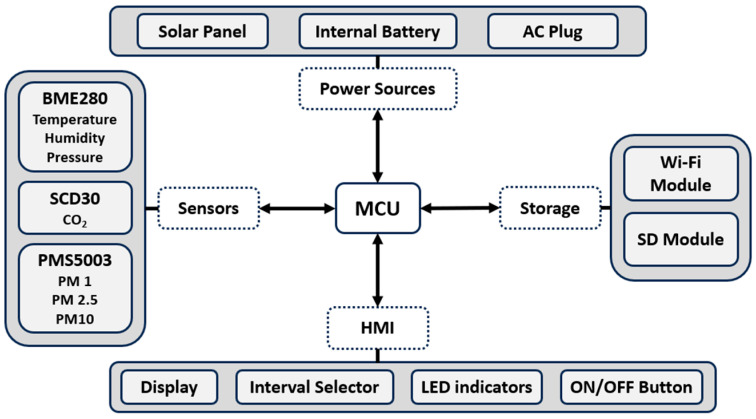
Modules for the open-source weather station (OSWS).

In summary, the five modules are shown in Figure 3. They are handled by a microcontroller unit (MCU); in this case, the STM32F103C8T6 was implemented.

For the sensing instrument, three sensors were chosen and are shown in Table 2 (these sensors can measure the variables within the conventional ranges):

For versatility purposes, three different power sources were chosen so the station could be placed in scenarios with several conditions:

- 100 W Solar Panel
- Li-ion Internal Battery
- AC Plug connection

Two storage options are available for the storage unit: local and remote. A 32GB SD card was added to the system for the first option. Since CSV is a flexible and compatible format, all the measurement values and timestamps were stored in this file.

On the other hand, the remote storage was managed through the modem ESP8266 with WiFi integrated; the values were sent to the ThingSpeak platform; this service stores each variable (i.e., temperature, relative humidity, PM 2.5) in a single channel, and the values collected can be exported via an API, or in CSV format. In addition, since this project aims to be open source, all the channels are open to the public. Table 3 shows the order in which the columns with the values are stored.

Finally, a human–machine interface was implemented so users could operate the OSWS efficiently. The elements included are:

- Display: to show the measurements on live.
- Display the on/off button.
- Interval selector button: to configure the measurement interval time.
- LED indicators: to indicate the device’s status.
- General on/off button.

Additionally, some other considerations were considered while designing the OSWS. Table 4 describes the solutions applied to accomplish the WMO recommendations.

**Figure 4:**
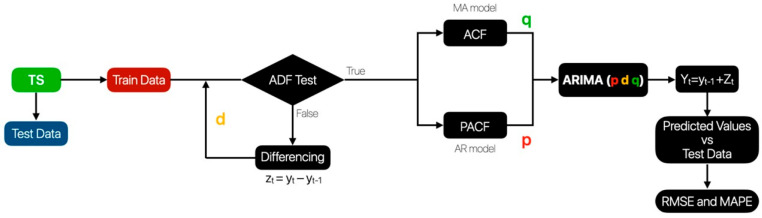
Methodology for forecast process.

The forecast process is the final stage of the methodology shown in the diagram in Figure 3. This part is described in detail in Figure 4.

As stated before, the forecast ARIMA model is univariate; hence, a single variable is considered to predict values from the same variable. For instance, if temperature values are to be expected, previous temperature values are considered in the model. Each set of values (temperature, PM 1, PM 2.5, CO_2_, etc.) is regarded as a time series since every measurement has a timestamp and is evenly spaced. This space between values is configured with the button that sets the measurement interval described in the human–machine interface.

**Figure 5:**
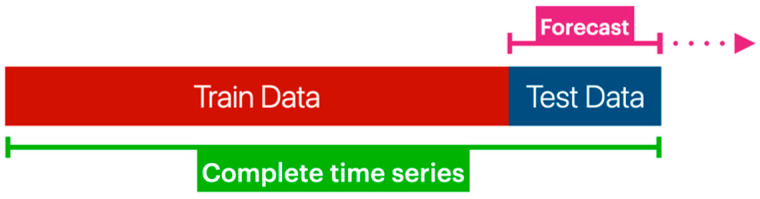
Time series divided into two parts.

The time series (marked as green in Figure 4) represents all the values collected from the device from a single variable. The following step was to split the dataset into train and test data. The first part trains the model; the other compares the predicted values and quantifies the model’s performance (Figure 5).

Furthermore, since the ARIMA model requires the time series to be stationary, the mean and standard deviation must be constant; therefore, a statistical test called Augmented Dickey–Fuller was implemented to assess this requisite. If the time series was non-stationary, a different method was applied to turn it into stationary. This method subtracts the previous value from the current value [33]. The following equation shows the first-order differencing process:

z_t_ = y_t_ − y_t−1_ (1)

where:

- z_t_ is the new time series storing the differences of the original time series.
- y_t_ is the current value of the original time series.
- y_t−1_ is the first previous value of the actual time series at time t.

Once this method is applied, the ADF test can be used to the time series Zt to determine if it is stationary. When this requisite is accomplished, the order for the model must be estimated. This model is made up of three parts: Autoregressive AR(p), Integrated I(d), and Moving Average MA(q); therefore, three parameters must be estimated: p, d, and q [34].

**Figure 6:**
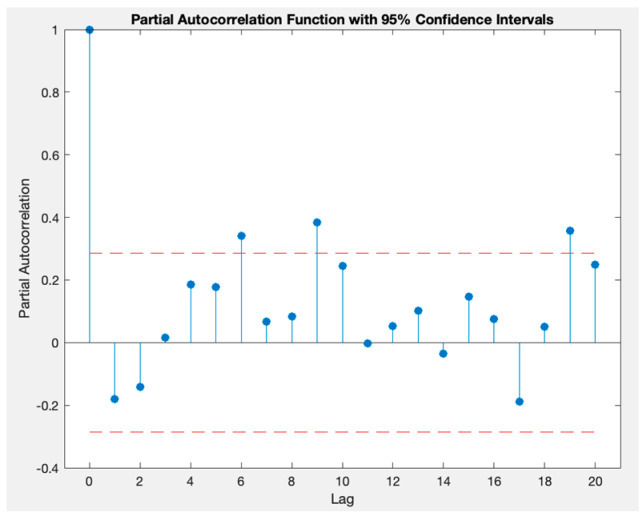
PACF plot of a temperature times series.

The “d” parameter represents the number of times the differencing method was applied to the time series. In the case of the “p” parameter, it is estimated with the partial autocorrelation function (PACF) plot; on the other hand, the “q” parameter is calculated with the autocorrelation function (ACF). In both cases, the lags considered for the model are those outside boundaries. For example, in the case of Figure 6, a PACF plot is illustrated; in that graph, the lags 6, 9, and 19 should be considered in the model [33,35,36,37]. The boundaries or horizontal lines represent the confidence intervals, which are statistically derived to help determine the significance of the partial autocorrelations at different lags.

### 3.2. Data Validation

The system proposed was tested on indoor and outdoor scenarios and compared against a reference instrument. The first scenario was inside an enclosed room, and the reference instrument was Schneider’s thermostat (ST), which measures only temperature and humidity and shows the live measurements through a display.

**Figure 7:**
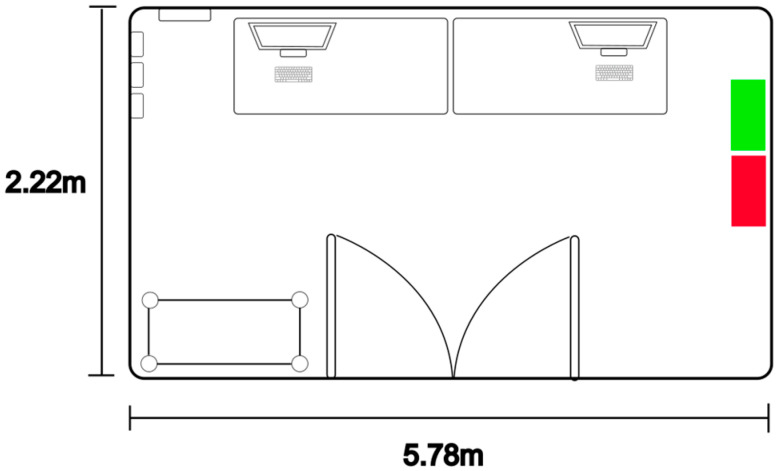
The layout of the measurement site.

Schneider personnel gave access to the site where the thermostat was placed for 1 h (from 12 pm to 1 pm); since photos were not allowed, Figure 7 shows a layout of the area. As illustrated, the proposed OSWS (red rectangle) was placed next to the thermostat (green rectangle) and was configured to have the same measurement interval, in this case, 30 s. It is essential to highlight that there were no windows, and the single door was closed; hence, there was no airflow. During this process, the OSWS was powered with an internal Li-ion battery.

For outdoor comparison, the OSWS was placed on a house roof exposed to the air for one day. The reference instrument was a Purple Air sensor 2.118 km away, which is considered a small distance, and the weather conditions.

**Figure 8:**
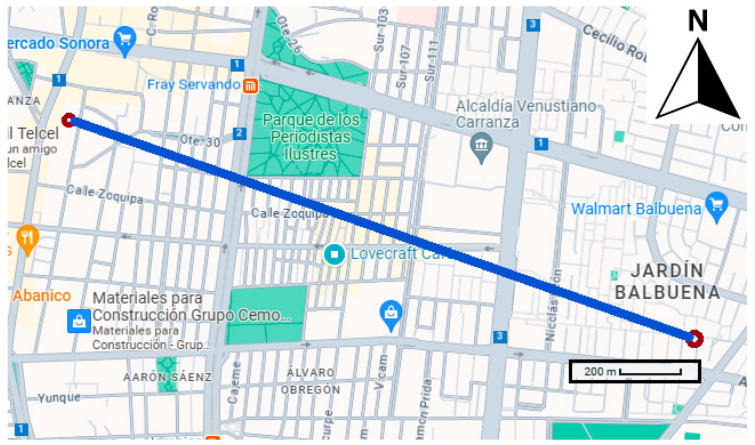
Distance between OSWS and Purple Air sensor.

Figure 8 shows the map and the distance the Google Maps app estimated. The purple point is where the weather station was placed, and the red point represents the Purple Air sensor. The line represents the distance between the OSWS and the Purple Air sensor. The red dots are the specific locations. 

The measuring time was 24 h (from 13th May at 01:30 to 14th May at 1:30), with a measurement time interval of 10 min. The data obtained from the OSWS were stored on an SD card for further analysis, and the data from the reference tool were downloaded from Purple Air´s website.

## 4. Results

### 4.1. Design Results

**Figure 9:**
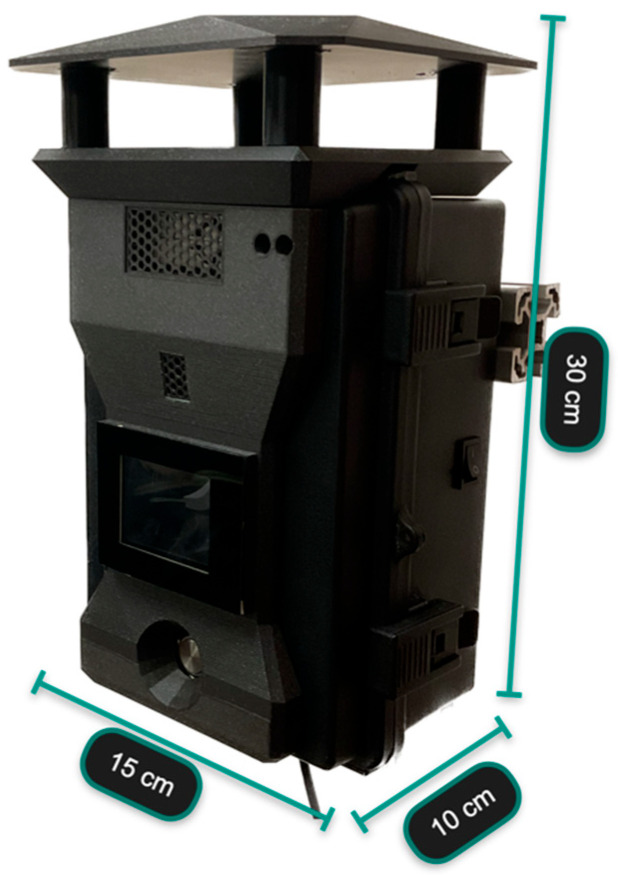
OSWS size measurements.

Following the WMO recommendations described earlier and inspired by the state-of-the-art projects, the OSWS prototype was developed. The final result is shown in Figure 9, illustrating the size measurements. All the electronic components that handle and manage the system are placed inside this case.

**Figure 10:**
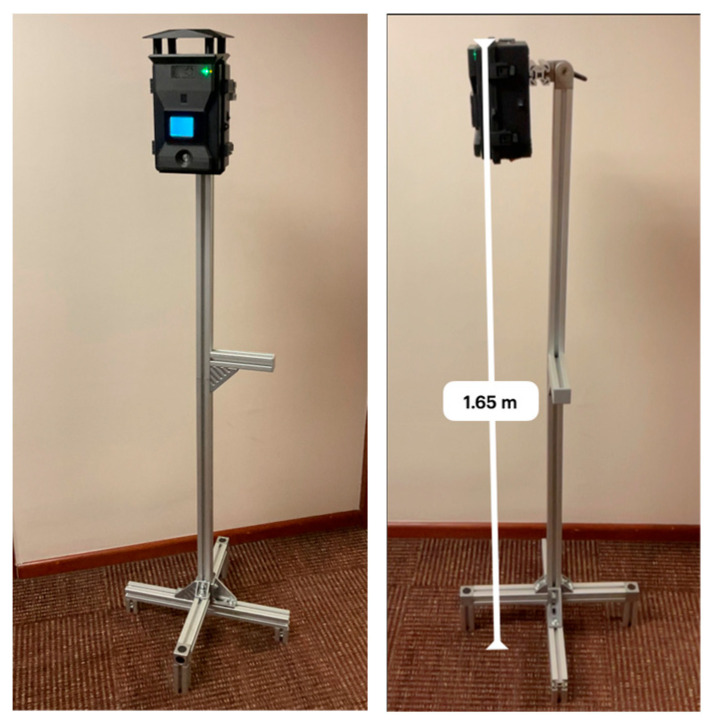
OSWS mounted in a 1.65 m height base.

Moreover, Figure 10 illustrates the proposed solution; the photos show the weather station attached to a 1.65 m aluminum base powered by the internal Li-ion battery.

**Figure 11:**
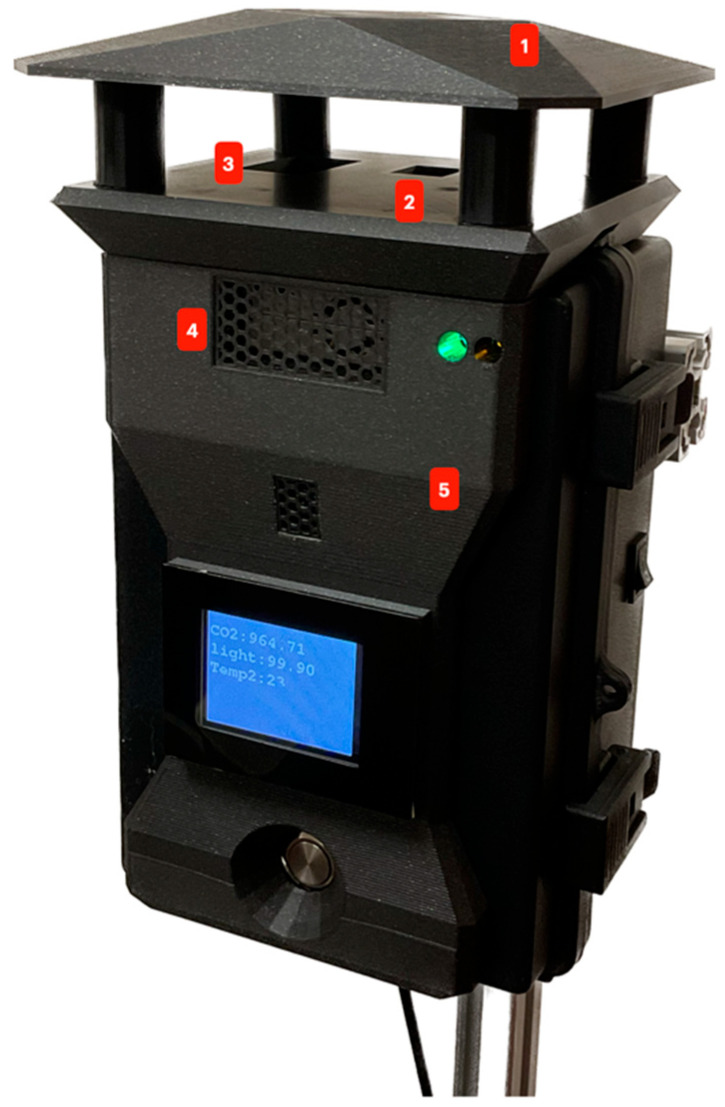
Sensor and protection for rain and sun.

Figure 3 shows three sensors in the sensing system that measure air temperature, relative humidity, atmospheric pressure, CO_2_ concentration, and PM 1, PM 2.5, and PM 10 concentration. According to the WMO, the sensors should be exposed to the air but protected from rain or direct sunlight. Figure 11 shows how the sensors are distributed along the case, which has IP65 protection, keeping the internal electronic components away from dust and water, and the accessory at the top of the device is designed to protect the sensors from rain and sun.

Figure 11 presents the following components:

1. Accessory for rain and sun.
2. BME280 measures temperature, relative humidity, and atmospheric pressure.
3. SCD30 for CO_2_ concentration measurement.
4. PMS5003 for PM (PM 1, PM 2.5, and PM 10) concentration.
5. IP65 case.

**Figure 12:**
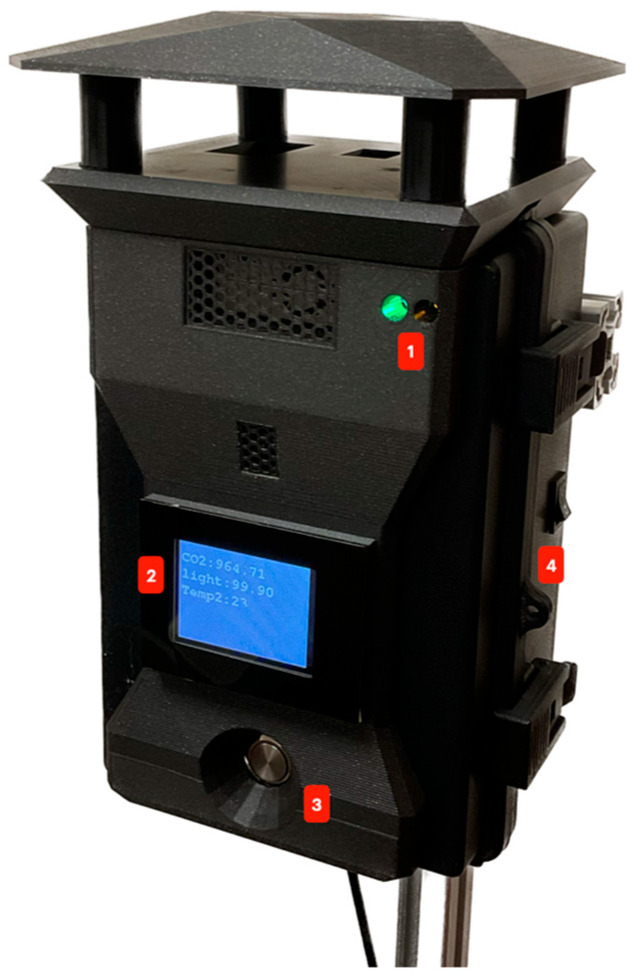
HMI implemented in the device.

**Figure 13:**
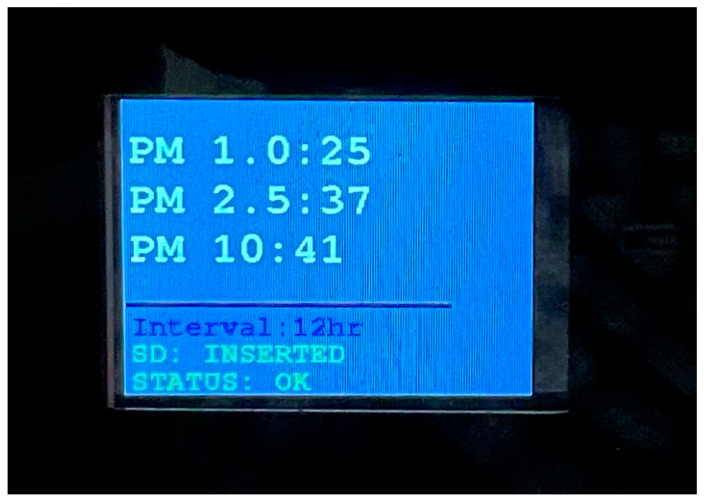
Display.

Moreover, the HMI, shown in the diagram in Figure 3, was implemented as illustrated in Figure 12, where:

1. Indicator LEDs. Green: if the system is measuring. Yellow: if there is some trouble with a sensor.
2. 2.4″ Display (Figure 13) showing:
  - Live measurements.
  - Interval selected.
  - SD status (inserted or removed).
  - Sensing platform status (measuring or not).
3. Interval selector: used to configure the interval measuring time:
  - 30 s
  - 1 min
  - 10 min
  - 30 min
  - 1 h
  - 12 h
  - 24 h
4. On/off button for the display.

**Figure 14:**
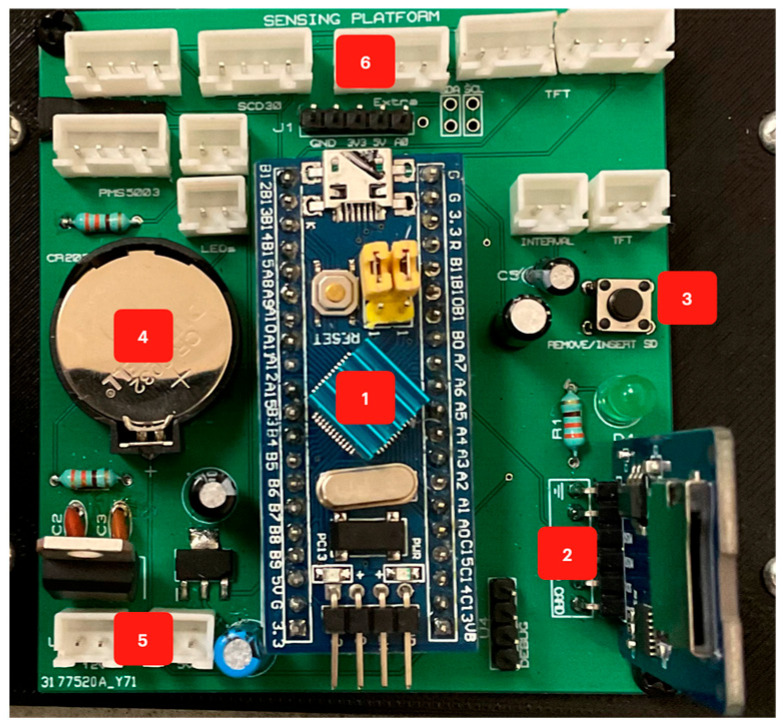
Main PCB.

**Figure 15:**
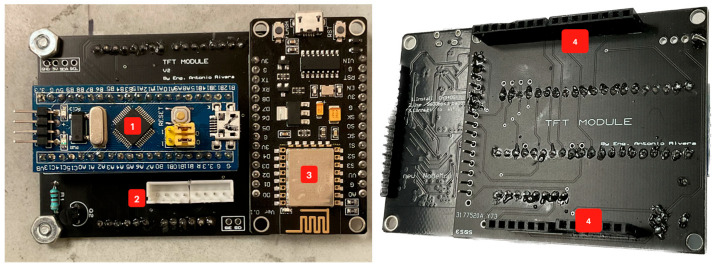
Second PCB.

Two microprocessors and a wireless board were installed to handle all the sensors and the HMI. This solution prevents overheating a single MCU and provides an easier way to modularize the sub-systems. Following this design, two PCBs were designed: main PCB (Figure 14) and second PCB (Figure 15).

The main PCB includes:

1. The main MCU that manages the sensors stores the data in the SD card, controls the timestamp obtained from the RTC, and sends the data shown through the display.
2. SD module controlled with SPI protocol.
3. Button for removing or inserting the SD safely.
4. 3V Coin battery to power the RTC even when the primary power source is disconnected.
5. Voltage regulators.
6. JST connectors for a secure component connection.

On the other hand, the second PCB includes:

1. MCU that manages the 2.4″ display and controls the WiFi board.
2. JST connector for communication with the main PCB.
3. WiFi board (ESP8266) that sends the data via the internet to ThingSpeak.
4. Headers to attach the display.

### 4.2. Ergonomics Assessment

Once the prototype was functional, it was evaluated in terms of ergonomics. It is a science focused on equipment design with a human-centered approach to reduce operator fatigue or discomfort considering physical, cognitive, social, organizational, and environmental factors [38,39]. Since this science focuses on improving design performance, the degree of usability must be quantified to accurately assess the ease of use of the designed prototype; therefore, several helpful methods exist for this purpose. For instance, keystroke-level model, link analysis, questionaries, checklist, hierarchical task analysis, layout analysis, and interview, among others [39]; the selection of the methods for the assessment depends on the project´s design stage in this case. the project has a functional and physical prototype. Therefore, the methods selected were observation for the usability link analysis and layout analysis for the ergonomics test [39].

The observation method was performed by giving the users the device and asking them to perform planned activities; in this case, six users were selected, and six different activities were established:

1. Move the station to a specific point.
2. Turn on the device.
3. Set the measurement interval time to 24 h.
4. Turn off the display.
5. Open the case to get access to the internal components.
6. Remove the SD card.

Only six users were selected since, when evaluating the usability of a product, it is enough for only five users to use the product because they will find more than 75% of the product issues. Using fewer users will lead to not covering most of these problems; meanwhile, using more than six users, repeated issues will be found, making the results meaningless [40].

Each user had ten points at the beginning of the test, and then each wrong action or asking of a question was penalized by subtracting one point each time; in addition, the time that the user took to complete all the activities was measured. Table 5 shows the average score of each activity performed by the six users and the average time. In general, the overall score was 8.7 regarding usability.

Regarding ergonomics, the layout and link analyses were performed simultaneously on the human–machine interface presented in Figure 12. The first method states that the components from the same category must be as close as possible. There were only two categories found: device control and power. On the other hand, the second method consists of establishing a sequence of actions that would be one of the most common sequences while using the device in a normal operational mode and tracing lines between the components following that order [39]. In this case, the sequence was:

1. Turn on the device.
2. Check the indicator LEDs.
3. Turn on the display.
4. Watch the display and the measurements.
5. Change the measuring interval time to a different value.

**Figure 16:**
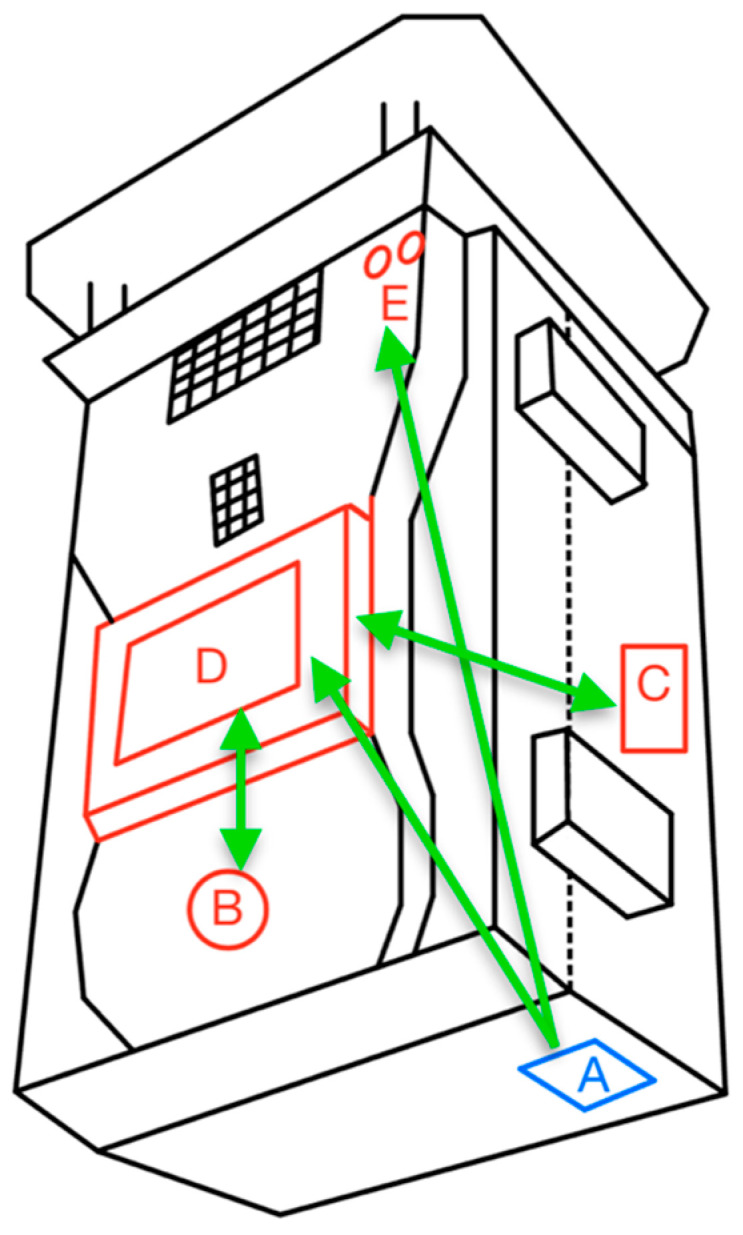
Layout analysis and link analysis.

Figure 16 depicts the two groups (red for device control and blue for power) and the lines (green) that follow the order established. The device control group includes the components labeled B, C, D, and E; the power group includes only component A.

Once the lines are traced, the method establishes that no line should cross with another to enhance operability and ergonomics. In this case, line A-E crosses line C-D, indicating that one or more components should be relocated.

**Figure 17:**
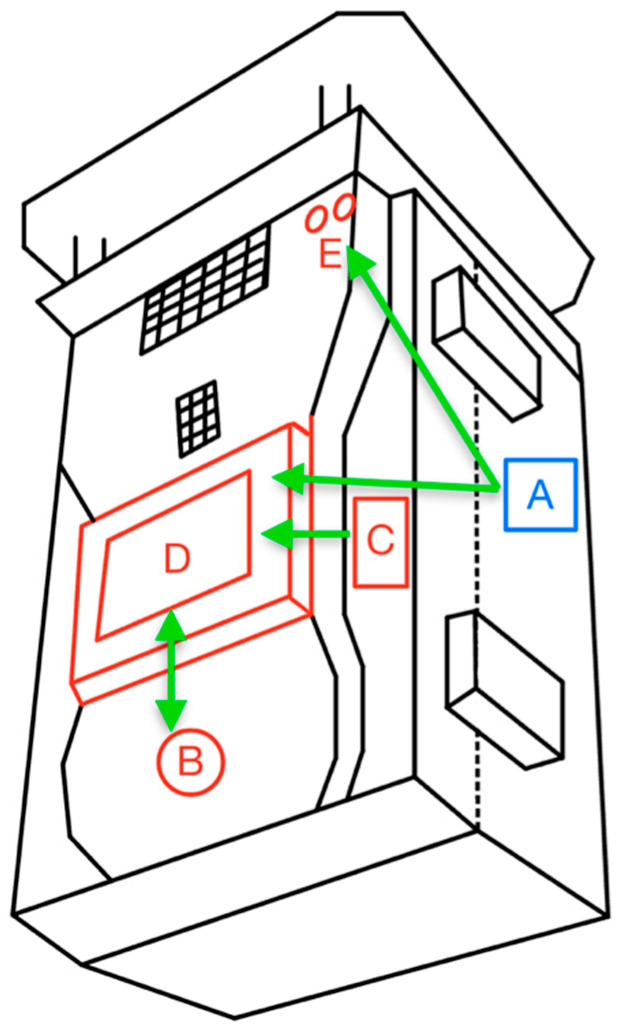
Components relocated.

Figure 17 shows the rearrangement of the components that satisfy the link analysis requirements. Furthermore, it can be observed that the four elements (B, C, D, and E) are now grouped and satisfy the layout analysis requirements.

### 4.3. Data Comparison

**Figure 18:**
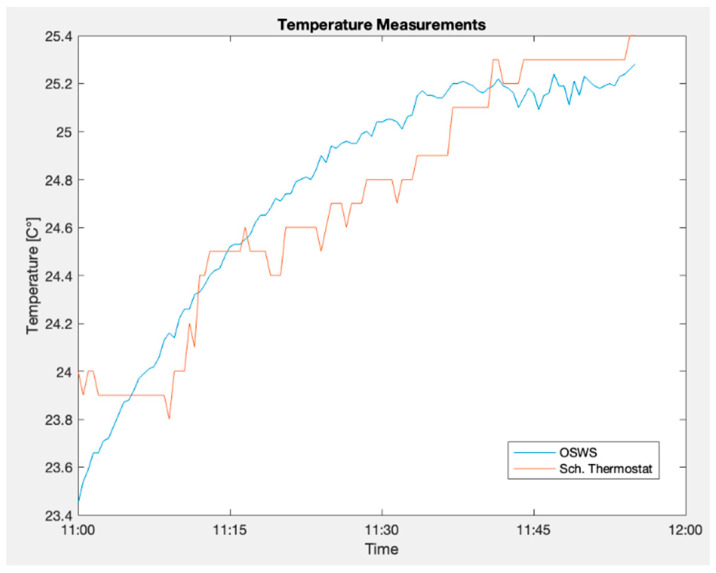
OSWS and reference device plot in an indoor scenario for temperature.

The first comparison was between the designed weather station and the Schneider Electric thermostat. As stated, the two devices were placed together, as shown in Figure 7. The data from the reference instrument were provided directly by Schneider´s personnel. Figure 18 shows the two signals; the blue line represents the measurements performed by the current proposal, and the orange one is the reference value. At first glance, the two signals seem to have the same direction, and a monotonic relationship can be inferred.

Statistical parameters were obtained to compare the two signals and determine their relationship. The first non-parametric value is the Spearman correlation, which indicates the strength of the association, a number between −1 and 1, and the direction of the association, which can be positive or negative [40]. Additionally, the root mean square error (RMSE) and mean absolute percentage error MAPE were calculated to determine the performance of the measurement process; these parameters are mainly focused on rating a forecast model’s accuracy, but they can be applied to quantify the total error of two signals.

**Figure 19:**
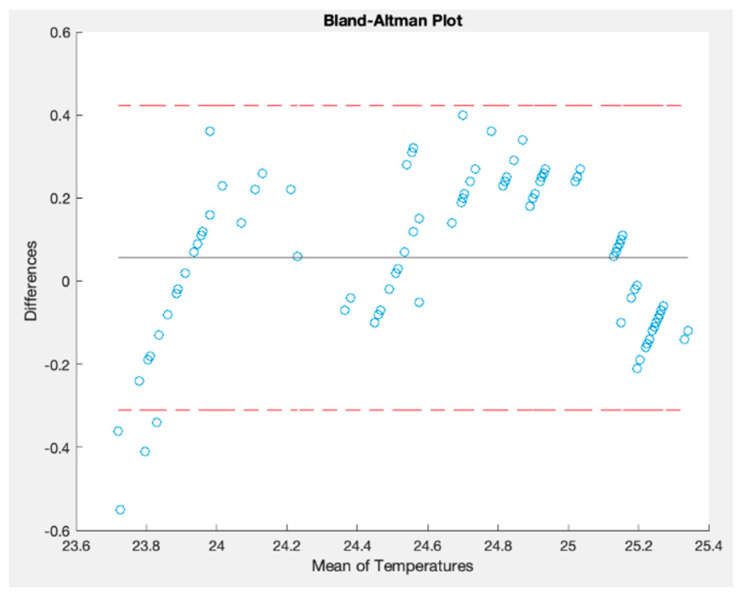
Bland–Altman plot for temperatures measured indoors.

Moreover, a Bland–Altman plot was generated to assess the agreement between these two signals. On the *y*-axis, the differences between the measurements are displayed; on the *x*-axis, the averages of the measurements are plotted. Each plot on this graph is a pair of measurements taken by the two instruments. Finally, this Bland–Altman method has two limits of agreement at ±1.96 times the standard deviation. Ideally, all the points should lie between these two limits, representing an agreement between them [41]. Figure 19 shows the Bland–Altman plot generated by comparing the time series.

This figure indicates a good agreement between these signals since most points are between the interval limits [41]. The differences between the paired values increase when the temperature is between 23.6 °C and 23.8 °C. Table 6 shows the values obtained for the other statistical parameters. The correlation parameter is a high value indicating a strong relationship between the two measurements [42]. In addition, RMSE and MAPE are low values, demonstrating that the OSWS has similar measurement values to Schneider´s thermostat.

**Figure 20:**
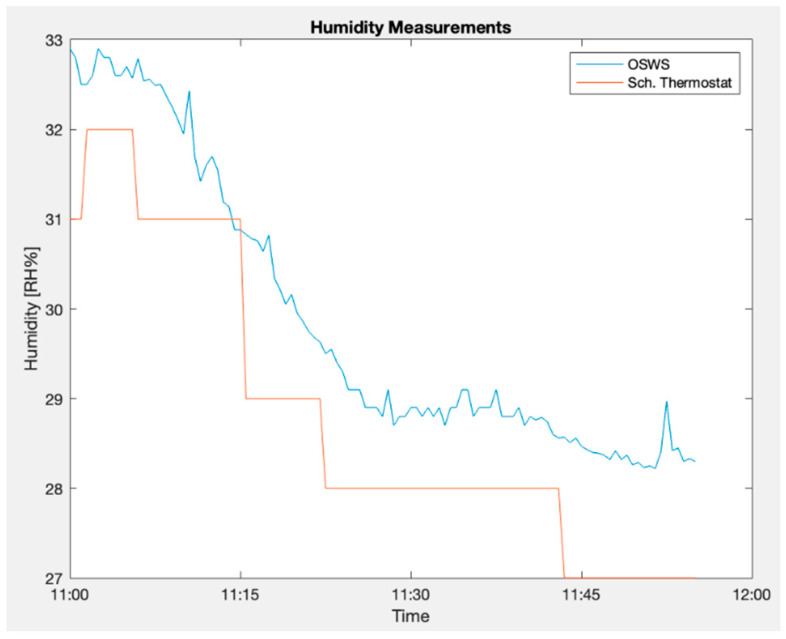
OSWS and reference device in an indoor scenario for relative humidity.

**Figure 21:**
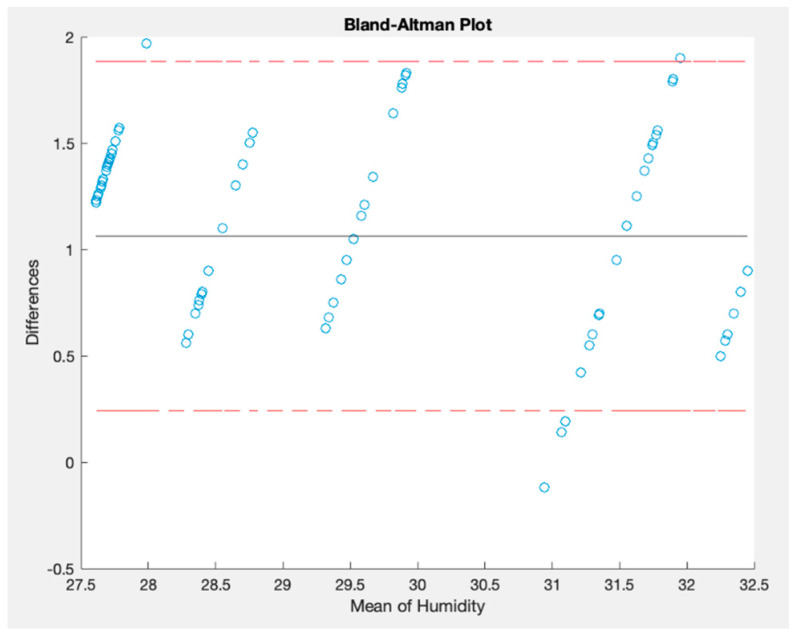
Bland–Altman plot for relative humidity measured indoors.

In assessing humidity measurements, Figure 20 illustrates a comparative analysis between the proposed OSWS and the Schneider’s thermostat over a specified time frame. Concurrently, Figure 21 presents the Bland–Altman plot, highlighting that most values reside within the stipulated boundaries. This pattern suggests a favorable level of agreement between the two measurements (air temperature and relative humidity) since most points are between the two red lines representing the limits of agreement in which 95% of the differences between the two measurements are expected to fall.

**Figure 22:**
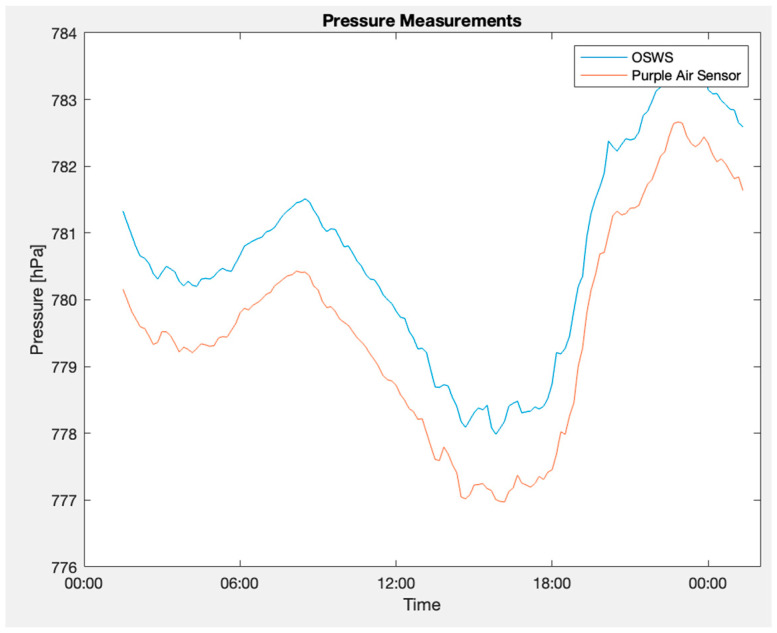
OSWS and reference device plot in an outdoor scenario for atmospheric pressure.

**Figure 23:**
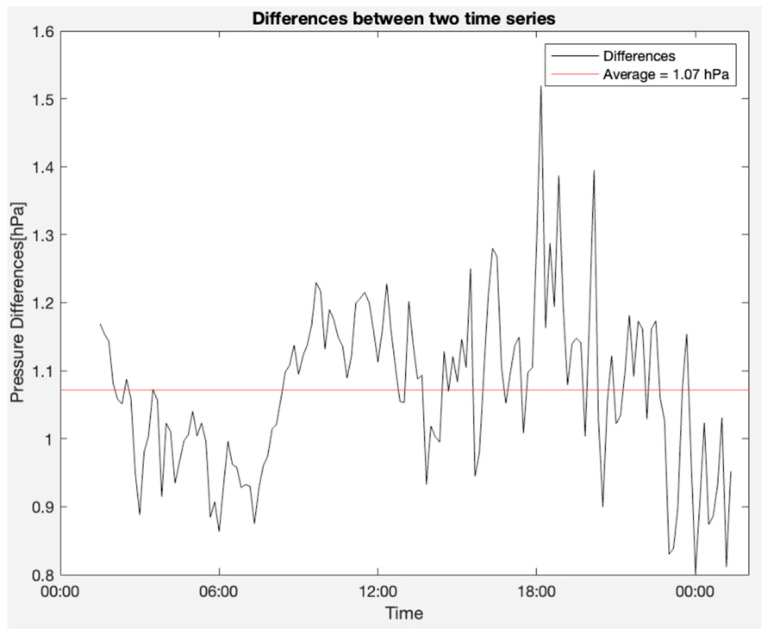
Differences between the two pressure time series.

**Figure 24:**
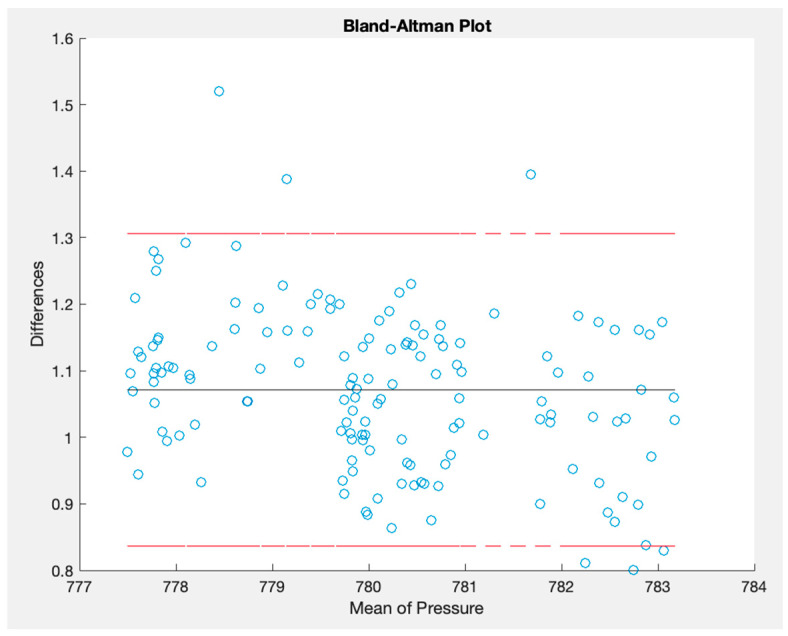
Bland–Altman plot for pressure measured outdoors.

The OSWS was juxtaposed with a Purple Air sensor located approximately 2.118 km away in the outdoor environment. The methodologies employed were consistent with those used in the indoor experiments. Comprehensive quantitative findings are enumerated in Table 7. Moreover, Figure 22 depicts the atmospheric pressure graphs where the upper blue line represents the signal from the proposed station. In contrast, the red line at the bottom corresponds to the reference sensor. Notably, the difference in readings between the two signals in Figure 22 could be attributed to the variance in altitude where each sensor was positioned. Figure 23 highlights the measurement discrepancy, revealing an average difference of 1.07 hPa. Figure 24 shows a Bland–Altman plot that affirms a notable alignment between the two signals, with most of the points nestled within the limits of agreement. Additionally, it is paramount to consider the inherent biases of the sensors, which can introduce measurement discrepancies. External noise, including environmental factors or electronic interference, can also influence the readings, affecting the system’s accuracy.

**Figure 25:**
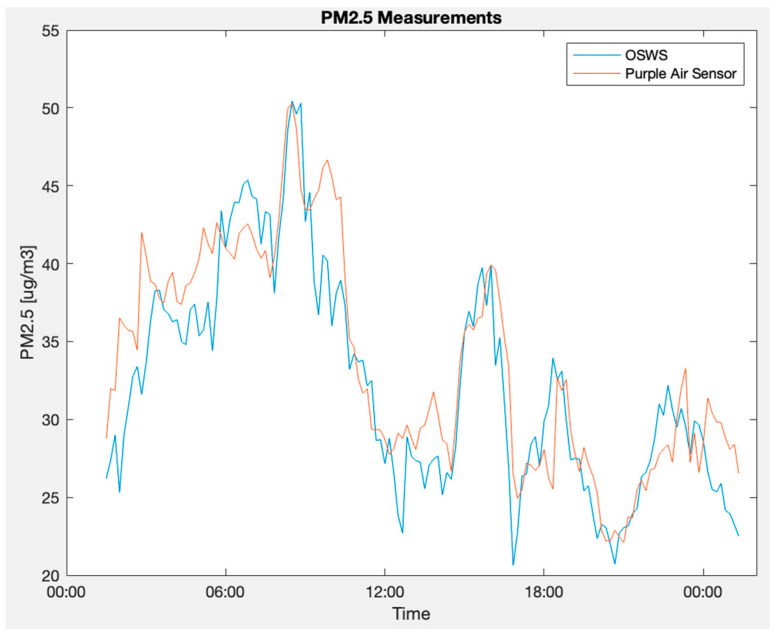
OSWS and reference device plot in an outdoor scenario for PM 2.5.

**Figure 26:**
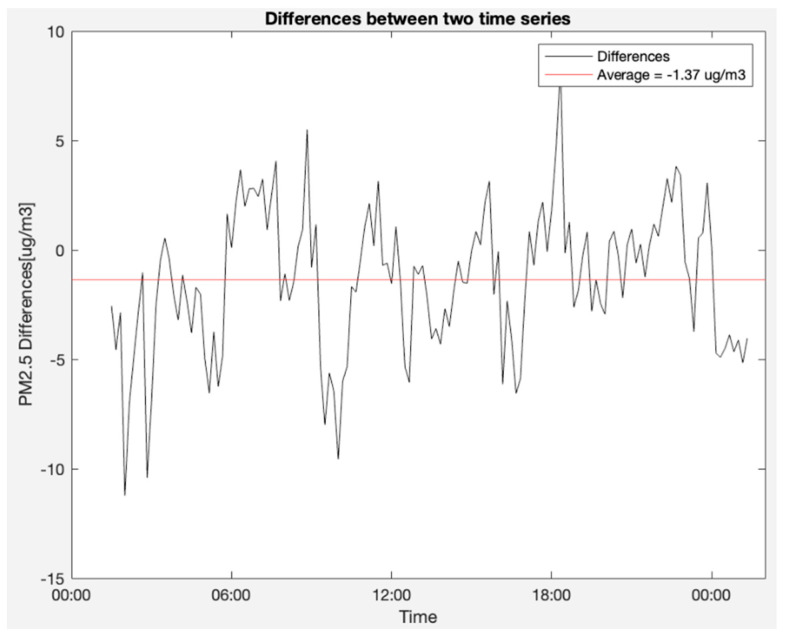
Differences between the two PM 2.5 time series.

**Figure 27:**
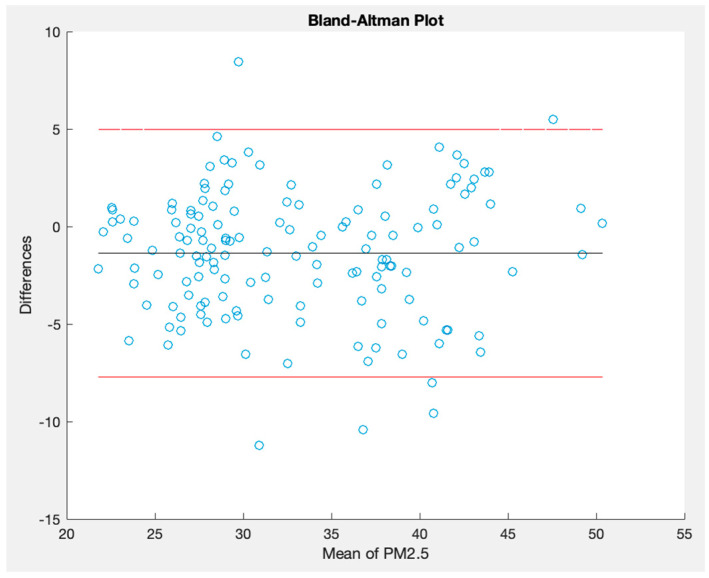
Bland–Altman plot for PM 2.5 measured outdoors.

Furthermore, concerning PM 2.5 measurements, Figure 25 captures the simultaneous readings from the OSWS and the reference device in an outdoor scenario. Figure 26 details the disparities between the two PM 2.5 time series, shedding light on periods of convergence and divergence. Finally, Figure 27 employs a Bland–Altman plot to visualize the agreement of PM 2.5 values measured outdoors between the OSWS and the reference device, reinforcing the consistency and reliability of the OSWS measurements.

These numbers provide essential information and insights about the data gathered. Regarding the Spearman correlation, all the data collected from the sensors are considered to have a robust positive correlation since all the Spearman correlation values are above 0.8 [40,42]. In the case of the RMSE parameter, all the measurements were below five units, and the MAPE was below 10%. It is essential to mention that the air temperature and relative humidity may be affected by the buildings near the OSWS and the house where the device was placed [14].

## 5. Discussion

### 5.1. Comparison with the Existing Literature

The introduction of the OSWS in the realm of weather monitoring marks a significant stride. To contextualize its place amidst the extensive literature on the subject, it is vital to delineate how the OSWS aligns with, deviates from, and potentially surpasses contemporary solutions. The core strength of OSWS lies in its commitment in democratizing weather data access using cost-effective sensors. Many monitoring systems in the current literature either prioritize accuracy over affordability or vice versa. However, the OSWS strikes a balance, ensuring that smaller communities and users are not left behind in the age of data-driven decision-making. This holistic approach offers a twofold advantage: it ensures affordability without compromising the essential quality of weather data.

Furthermore, real-time data sharing and monitoring capabilities distinguish the OSWS from many conventional systems. In sectors where swift responses to changing weather conditions are paramount, the immediacy of data becomes indispensable. The potential of OSWS to create a connected network of weather stations exponentially amplifies this benefit, enhancing data accessibility and utility across regions.

To encapsulate the distinctions and similarities between the OSWS and other research endeavors, the comparative Table 8 offers a concise quantitative perspective, aiding in discerning the unique propositions the OSWS offers. This table shows the root mean square error that each station has against a professional measuring tool in an indoor scenario and whose values are considered as the reference.

### 5.2. Impact

The revolutionary aspect of the OSWS is its democratization of weather data through low-cost sensors, facilitating accessible and affordable weather monitoring. By enabling real-time data sharing, the OSWS fosters timely decisions, which is crucial for sectors sensitive to weather fluctuations. Apart from real-time weather monitoring, the broader vision behind the OSWS extends to its application across diverse sectors like agriculture and urban development. Its educational utility, offering a tangible tool for learners, further underscores its multifaceted applicability.

The OSWS, with its potential for regional weather analysis and integration from multiple units, lays the groundwork for enhanced predictive capabilities, with notable relevance in sectors relying heavily on accurate weather forecasts.

### 5.3. Limitations and Future Directions

While the OSWS heralds a transformative shift in weather monitoring, certain limitations prevail. These encompass the system’s precision, which, while consistent, might be inherently limited due to its cost-effective approach. Another pivotal concern is data transmission and security, especially in regions with unreliable internet connectivity.

Future endeavors related to the OSWS ought to emphasize:

1. Secure and dependable data transmission, particularly in remote settings.
2. ExplorING alternative data transmission channels and offline data logging/retrieval solutions.
3. Strengthening data security measures to preserve data integrity during transmission and storage.
4. Investigating durable sensors and materials to withstand environmental challenges, reinforcing the OSWS’s reliability.

## 6. Conclusions

The OSWS offers a groundbreaking approach to weather monitoring, harnessing low-cost sensors to overcome challenges posed by high costs and the need for expert personnel inherent in existing technologies. Its capacity for real-time monitoring and forecasting of pivotal environmental variables using the ARIMA model stands utmost for agriculture, transportation, and air quality monitoring. Its open-source nature further amplifies data accessibility, fostering informed decisions essential in our current climate-changing era.

The design and implementation of the OSWS laid the groundwork for the future evolution of cost-effective and efficient monitoring systems. As we move ahead, focusing on enhancing data transmission security, diversifying communication interfaces, and enduring diverse environmental conditions will be pivotal in refining the OSWS, aiming to bolster environmental sustainability and foster informed decision-making amidst escalating ecological challenges.

## Figures and Tables

**Table 1:** State-of-the-art based on related projects.

Ref.	Variables	IN/OUT	DY	ST	WT	MIT	PS	Aut. B	Op-S	IW	Design	OS	GPS	Forecast	VR	MCU	C
[22]	Temperature and RH: SHT75 Solar radiation: SP110	Both: Ip65 Box Solar radiation shield 2 m mast	No	RPI SQlite and send to ThingSpeak	Xbee RF	Configurable between 30 s to 5 min	Solar panel with battery 10 Ah AC	24 h	No	No	Compact	Raspbian OS	No	Yes Nearest-neighbors and RBF ANN 4 h horizon	No	RPi	300 mA Normal mode 150 mA without ethernet, USB, etc. 60 mA Xbee
[28]	NO_3_, O_3_: NO2A43F/OXA431 PM 10, PM 2.5: SDS011 Temperature, humidity, atmospheric pressure	Both: Ip 67	No	SD Card	Bluetooth	25 s fixed	Internal rechargeable battery AC	8 h	No	No	Compact	No	Yes	No	Yes Against professional WS ARPA Lazio	Arduino	Not specified
[19]	PM 2.5, temperature, humidity	No	No	SD Card	No	Not specified	Alkaline 9 V battery	Not specified	No	No	Compact	No	Yes	No	No	Arduino	Not specified
[29]	NO_2_, SO_2_, CO_2_, CO, PM 2.5, temperature, and humidity	Both	No	No local storage. Only Server connection	Lora	10 s fixed	Solar panel	Not specified	Yes	No	Compact	No	No	No	Yes Against Aeroqual	Arduino	Not specified
[20]	Air temperature, rainfall, wind speed, wind direction, relative humidity, atmospheric pressure	Both	No	2 GB SD Card	SIM900 GPRS	1 min fixed	55 W Solar panel	Not specified	No	No	Compact	No C code	No	No	No	Arduino	Not specified
[21]	Soil humidity Soil temperature Air temperature Air humidity Atmospheric pressure Wind vane Wind direction Rain gauge	Both	No	16 GB SD Card	ESP32 WiFi	1 hr fixed	Solar panel 55 W Li-ion battery	1.5 months Only 1 measurement per hour	No	No	Compact	No C code	No	No	No	RPi Pico	Not specified
This work	Air temperature, relative humidity, atmospheric pressure, CO_2_, PM 1, PM 2.5, PM 10	Both IP65 box 1.6 mast	Yes 2.4″ TFT	32 GB SD Card ThingSpeak server	ESP8266 WiFi	Configurable: 30 s, 1 min, 10 min, 30 min, 1 h, 12 h, 24 h.	Solar panel 100 W AC Internal Li-ion battery	48 h in full mode	Yes Exportable data in CSV API integration	Yes Aluminum foil	Compact All-in-one	No C code	No	Yes ARIMA model	Yes Against Schneider thermostat	STM32 ARM-M3	230 mA average in full mode

IN/OUT: Indoors and Outdoors. DY: Display. ST: Storage. WT: Wireless Technology. MIT: Measurement Interval Technology. PS: Power Sources. Aut. B: Autonomy with Battery. Op-S: Open-source. IW: Isolated Wires. OS: Operative System. VR: Validation with Reference. PC: Power Consumption.

**Table 2:** Sensors incorporated in the weather station.

Sensor	Variables Measured	Unit	Accuracy	Response Time
BME280 [30]	Air Temperature Relative Humidity Atmospheric Pressure	°C % RH hPa	±0.5 °C ±3% RH ±1 hPa	1 s
SCD30 [31]	CO_2_ Concentration	ppm	±30 ppm	<20 s
PMS5003 [32]	Particulate Matter Concentration	μg/m^3^	±10%	<10 s

**Table 3:** Order of the columns stored in a CSV file.

Hour	Second	Date	Month	Year	AT	RH	AP	CO2	PM 1	PM 2.5	PM 10
[h]	[s]	[DD]	[MM]	[YY]	[°C]	[RH%]	[hPa]	[ppm]	[μg/m^3^]	[μg/m^3^]	[μg/m^3^]

AT = Air Temperature. RH = Relative Humidity. AP = Atmospheric Pressure. CO_2_ = Carbon Dioxide.

**Table 4:** Recommendations and proposed solutions.

Recommendation (Components)	Implemented Solution (Components)
Data acquisition (sensing instruments)	Specialized low-cost sensors
Plug-and-play connectors	JST connector
CPU	STM ARM Cortex-M3
Modem	ESP8266 module WiFi for one-way communication
RTC clock	Internal RTC clock
Power Supply	Internal battery PV module AC plug
Sampling one minute at least	Configurable measurement interval 30 s, 1 min, 10 min, 30 min, 1 h, 12 h, 24 h
Available memory for hundreds of days	32 GB SD Card up to 10 years for measurements each 30 s
**Recommendation (Design)**	**Implemented solution (Design)**
Easy component replacement	Plug-and-Play sensors, components, display, and MCU.
Stand-alone AWS	All-in-one design
Visual indicators	s
Extreme weather-proof case	IP65 case to cover all the components
Sensors exposed to air but covered from direct sunlight	3D PLA case to protect the sensors exposed to air
Supporting structure between 1.25 m and 2 m	1.6 m height mast

**Table 5:** Observation method results for the usability test.

Activity	Score Average	Average Time
Move the station to a specific point	9.5	4:14 min
Turn on the device	9.5	
Set the measurement interval time to 24 h	9	
Turn off the display	8.8	
Open the case to get access to the internal components	8.6	
Remove the SD card	6.7	
Average	8.7	

**Table 6:** Statistical parameters for temperature measurements.

Spearman Correlation	RMSE	MAPE
0.96	0.19 °C	0.67%

**Table 7:** Statistical parameters for measurements in an indoor scenario.

Variable	Spearman Correlation	RMSE	MAPE
Air Temperature	0.97	1.62 °C	7.01%
Relative Humidity	0.81	4.0 %RH	8.4%
Atmospheric Pressure	0.99	1 hPa	0.14%
PM 1	0.86	2.45 μg/m^3^	8.7%
PM 2.5	0.87	3.5 μg/m^3^	7.9%
PM 10	0.87	4.6 μg/m^3^	8.9%

**Table 8:** Comparative table between this OSWS and other projects in RMSE.

Reference	Temperature RMSE [°C]	Humidity RMSE [%RH]	Atmospheric Pressure RMSE [hPa]	PM 1 RMSE [μg/m^3^]	PM 2.5 RMSE [μg/m^3^]	PM 10 RMSE [μg/m^3^]
This OSWS	1.62	4	1	2.45	3.5	4.6
[20]	0.99	4.16	0.53	*	*	*
[29]	1.5	1.3	*	*	11	*
[21]	1.37	*	*	*	*	*
[22,28]	*	*	*	*	*	*

* Indicates that the variable was not compared with a reference.

## Data Availability

Data are contained within the article.
